# Acute presentation of a giant intrathyroidal parathyroid adenoma: a case report

**DOI:** 10.1186/s13256-016-1078-1

**Published:** 2016-10-19

**Authors:** Stephanie Rutledge, Michele Harrison, Martin O’Connell, Tadhg O’Dwyer, Maria M. Byrne

**Affiliations:** 1 Department of Endocrinology, Mater Misericordiae University Hospital, Eccles Street, Dublin 7, Ireland; 2 Department of Pathology, Mater Misericordiae University Hospital, Eccles Street, Dublin 7, Ireland; 3 Department of Radiology, Mater Misericordiae University Hospital, Eccles Street, Dublin 7, Ireland; 4 Department of Ear, Nose and Throat Surgery, Mater Misericordiae University Hospital, Eccles Street, Dublin 7, Ireland

**Keywords:** Parathyroid adenoma, Hypercalcemia, Parathyroidectomy, Primary hyperparathyroidism, Radionuclide imaging, Hungry bone syndrome, Case report

## Abstract

**Background:**

We report the case of a giant intrathyroidal parathyroid adenoma weighing 59 g in a young woman presenting acutely with severe hypercalcemia requiring correction and adequate preoperative management prior to surgery. Parathyroid adenomas account for 85 % of cases of primary hyperparathyroidism. Those weighing more than 3.5g are classified as giant parathyroid adenomas. There are only 25 cases of parathyroid adenomas weighing over 30g reported in the literature.

With the wide availability of biochemical screening tests in Western countries, mildly elevated calcium levels are often discovered incidentally. Our case is unusual for the extreme level of hypercalcemia, the patient’s young age, and the weight of the adenoma, particularly in a developed country.

**Case presentation:**

A 21-year-old Irish woman presented with a 3-week history of an enlarging right-sided neck mass. There was no dysphagia, stridor, or symptoms of hyperthyroidism or hypercalcemia. On examination, there was a firm painless swelling in the right lobe of her thyroid. Her thyroid function tests were normal. Corrected serum calcium was markedly elevated at 3.96 mmol/L with hypophosphatemia of 0.35 mmol/L. She was treated with bisphosphonates and fluids administered intravenously. Her parathyroid hormone level was over 20 times the upper limit of normal. Ultrasound revealed a solid and cystic nodule in the lower pole of the right lobe of her thyroid. Parathyroid scintigraphy demonstrated a 5×4 cm lesion which concentrated tracer.

A right-sided parathyroidectomy, right thyroid lobectomy, and level VI neck dissection were performed. An encapsulated multiloculated solid cystic mass weighing 59 g was removed. There was no definite infiltration of the capsule and MIB1 count was low at 1 % thus the specimen lacked the diagnostic features of carcinoma. On the third postoperative day, hungry bone syndrome developed and calcium replacement administered intravenously was required. At 1-year postoperative, she was weaned off calcium and alfacalcidol. A follow-up ultrasound showed unremarkable residual thyroid.

**Conclusions:**

Any patient with an isolated hypercalcemia warrants a thorough work-up. Hungry bone syndrome is a potentially avoidable condition; thus the clinician should be highly attuned to the risk of hungry bone syndrome post-parathyroidectomy, which correlates with the weight of the adenoma resected.

## Background

Primary hyperparathyroidism (PHPT) affects 22 per 100,000 people per year with a mean age of onset of 56 years and a female preponderance of 3:1 [1]. The incidence in patients aged 12 to 28 years is less than 5 % [2]. Solitary parathyroid adenomas account for 80 to 85 % of cases of PHPT. Less common causes include multiple adenomas, parathyroid hyperplasia, and parathyroid carcinoma.

The normal parathyroid gland weighs 50 to 70 mg. The median weight of parathyroid adenomas is 600 mg [3, 4]. Adenomas weighing more than 3.5 g [3] are classified as giant parathyroid adenomas (GPAs). In one series of 300 patients with PHPT, 5 % of resected adenomas weighed more than 3.5 g [3]. The most frequent etiological association with GPA is irradiation, as seen in Japanese survivors of an atomic bomb [5]. In 2009, there had only been 16 cases of adenomas weighing over 30 g reported in the literature [6]. A literature search of the past 5 years revealed another nine cases of adenomas weighing over 30 g. Our patient’s adenoma weighed nearly 59 g.

The largest adenoma described in the literature to date weighed 145 g [7] in a 63-year-old woman with a 3-month history of headache and fatigue; corrected serum calcium was 3.3 mmol/L. Another unusual case was an 85-year-old woman from rural Ireland who presented with stridor and was found to have a 110 g parathyroid adenoma [8].

## Case presentation

A 21-year-old Irish woman presented to an outside institution with a 3-week history of an enlarging right-sided neck mass. She had no significant medical history and was taking no medications. There was no dysphagia or stridor and she denied symptoms of hypercalcemia, hyperthyroidism, fevers, or weight loss. She did report mild constipation and two episodes of presyncope over the preceding weeks which were precipitated by exercise.

Her family history was notable for her maternal grandmother who died of sudden cardiac death and two maternal aunts with hyperthyroidism and goitres. Her mother had a duplex kidney system and was diagnosed with hypertension at the age of 23, necessitating four anti-hypertensive agents.

**Fig. 1:**
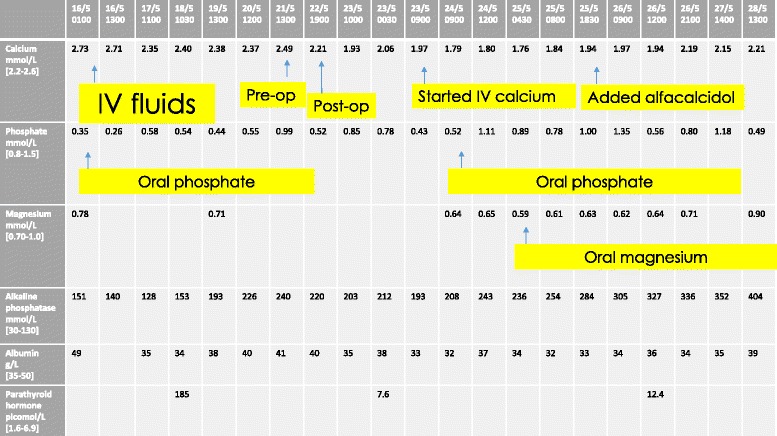
Demonstration of the decline in serum calcium with fluids and bisphosphonates administered intravenously. This figure also depicts the immediate fall in serum calcium, phosphate and magnesium following parathyroidectomy. *IV* intravenous

**Fig. 2:**
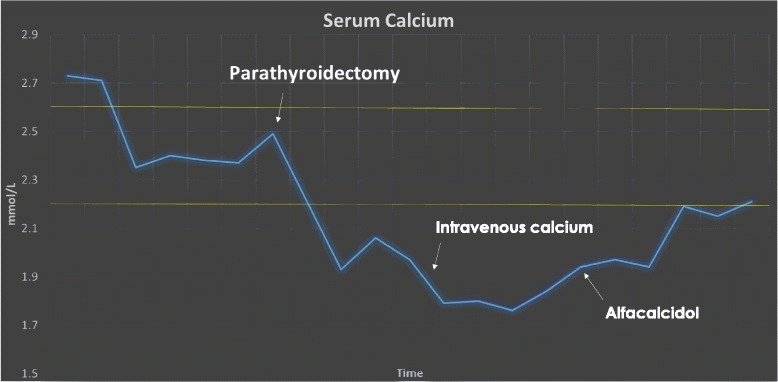
This graph demonstrates the immediate fall in serum calcium following parathyroidectomy

On examination, there was a firm painless swelling in the right lobe of her thyroid with no palpable lymphadenopathy. She was tachycardic with a heart rate (HR) of 112 but not hypotensive with blood pressure (BP) 116/80. Thyroid and renal function tests were normal: her serum creatinine was 64 mmol/L (44 to 86) and her estimated glomerular filtration rate (eGFR) was 124 mL/minute/1.73 m^2^ (normal ≥90 mL/minute/1.73 m^2^). However, her corrected serum calcium was markedly elevated at 3.96 mmol/L (2.2 to 2.6) with a hypophosphatemia of 0.35 mmol/L (0.80 to 1.5). Her albumin was 49 g/L (35 to 50). She was admitted acutely for fluids and bisphosphonates administered intravenously. Her calcium responded quickly, falling to 2.46 mmol/L within 3 days (Figs. 1 and 2). An ultrasound of her thyroid revealed a solid and cystic nodule in the lower pole of the right lobe of her thyroid measuring 5×4 cm. This was initially presumed to be a thyroid nodule and she was discharged with a scheduled out-patient appointment for thyroid fine-needle aspiration. However, 2 days later her parathyroid hormone (PTH) level was found to be over 20 times the upper limit of normal: 138.4 pmol/l (1.6 to 6.9). The PTH assay used was a second-generation Roche Platform electrochemiluminescence immunoassay “ECLIA” which measures intact PTH.

Six days later she was admitted to our institution. Her corrected calcium had risen again and now measured 2.73 mmol/L. Her vitamin D level was low at 35 nmol/L (<50) and her alkaline phosphatase was 143 IU/L (30 to 130). Her alkaline phosphatase levels rose over 5 days to 240 IU/L (30 to 130). A chest radiograph showed no osseous abnormalities.

The differential diagnosis included parathyroid adenoma, parathyroid hyperplasia, and parathyroid carcinoma. Multiple endocrine neoplasia type 1 (MEN1) was also a possibility, particularly given her young age.

**Fig. 3:**
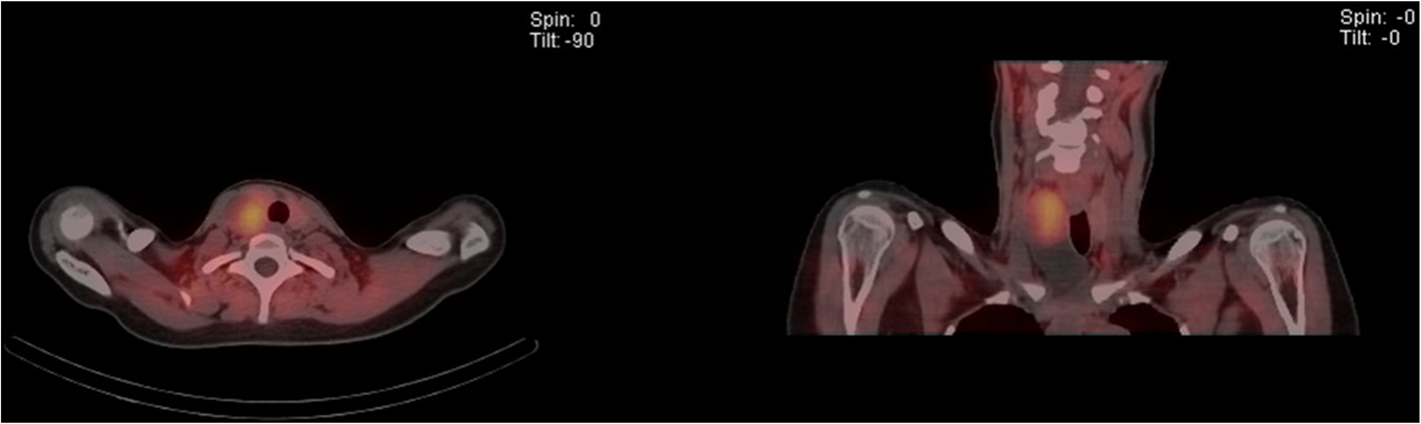
Single-photon emission computed tomography/computed tomography image at 2.5 hours post-radiotracer injection (Tc99m sestamibi) demonstrating a large lesion in the right lobe of the thyroid

Parathyroid scintigraphy (Tc99m sestamibi scan) demonstrated a lesion posterior to the right lobe of her thyroid which concentrated tracer and measured 7 to 10 cm in maximum length (Fig. 3). Her urinary calcium excretion was elevated at 9 mmol/24 hours (2.5 to 7.5/24 hours). Her prolactin level was normal, as were her urinary catecholamines and metanephrines.

**Fig. 4:**
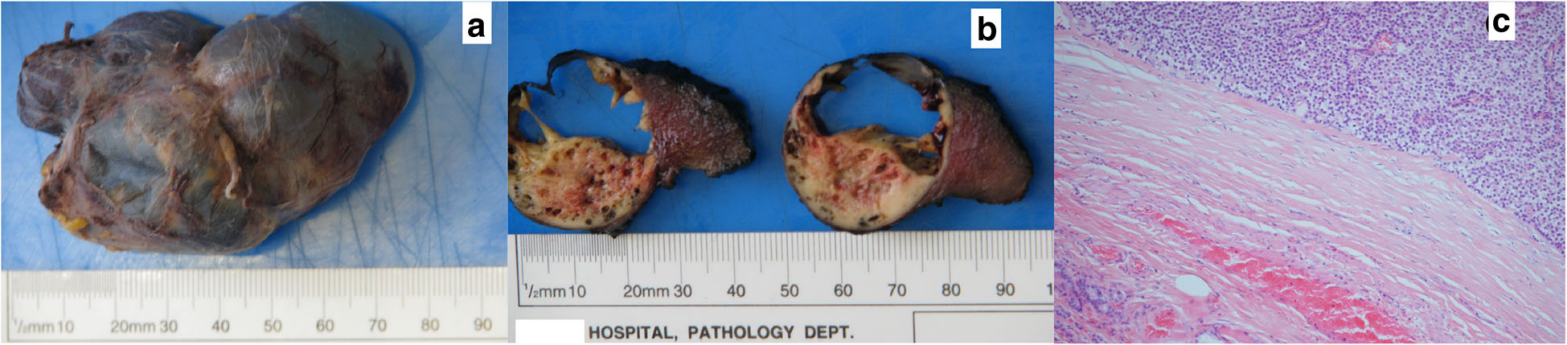
Giant parathyroid adenoma. **a** Gross specimen following resection. **b** Cross-sections of the multiloculated cystic mass. **c** Adenomatous cells under microscopy with adjacent normal thyroid tissue

Following surgical planning and adequate preoperative work-up, a right-sided parathyroidectomy, right thyroid lobectomy, and level VI neck dissection were performed. A level VI neck dissection was performed as there was a strong clinical suspicion of parathyroid carcinoma in view of the size of the enlarging lesion and her PTH levels. Her PTH was measured 4 days prior to surgery: 185 pmol/l (1.6 to 6.9). On postoperative day one, her PTH level had fallen to 7.6 pmol/l (1.6 to 6.9). An encapsulated multiloculated solid cystic mass measuring 80×55×30 mm and weighing 58.8 g was removed (Fig. 4a, b). There were focal areas of hemorrhage within the specimen. This was an atypical parathyroid adenoma due to its size and due to the presence of some cells and groups of cells in the capsule. There was no infiltration of the capsule meaning that the tumor cells did not extend beyond the capsule into the surrounding tissue. There was no perineural or vascular invasion and MIB1 count was low at 1 %; thus, the specimen lacked the diagnostic features of carcinoma (Fig. 4c). All ten dissected lymph nodes were free from tumor.

Her corrected serum calcium fell precipitously to a nadir of 1.76 mmol/L on the third postoperative day (Fig. 1). She developed tingling in her hands and feet and calcium replacement administered intravenously was required. Her alkaline phosphatase levels rose from 240 IU/L (30 to 130) preoperatively to 404 IU/L (30 to 130) on the sixth postoperative day. Her phosphate fell to a nadir of 0.43 mmol/L (0.80 to 1.5) and her magnesium fell to 0.59 mmol/L (0.7 to 1.0). These were both replaced orally. She was discharged well on the sixth postoperative day on calcium administered orally and alfacalcidol (1α-hydroxyvitamin D3). At 1-year postoperative, she is asymptomatic and has been weaned off both agents. Her serum calcium and PTH are within normal range and a follow-up ultrasound showed unremarkable residual thyroid.

## Discussion

Before transfer to our center, this patient was suspected to have a thyroid nodule and was scheduled for thyroid fine-needle aspiration. Fortunately a PTH level was checked and the correct diagnosis was reached. This case highlights that an intrathyroidal parathyroid adenoma could easily be mistaken for a thyroid nodule.

In the past, patients were more likely to present with extreme hypercalcemia and profound skeletal disease. With the availability of biochemical screening tests in Western countries, patients are now more likely to present with minor symptoms and mildly elevated serum calcium levels which are often discovered incidentally. Our case is unusual for the level of hypercalcemia, the acute presentation, the patient’s young age, and the weight of the adenoma, particularly in a developed country such as Ireland. The cystic mass we have described is very uncommon. Cystic parathyroid lesions make up less than 0.01 % of all neck masses [9]. They may be pure retention cysts or created by degeneration of an adenoma or carcinoma. In our case there was possibly partial cystic degeneration of the gland because of its huge size and probable long-standing nature, although the histopathological sections showing cysts did not reveal cystic degeneration [10]. A palpable neck mass is extremely rare in PHPT [11].

Several case series have supported the hypothesis that GPAs represent a distinct clinical entity with specific genomic aberrations [3, 12]. When compared to patients with parathyroid adenomas, the GPA group contains a higher relative number of male cases and cases of single gland disease (versus multiglandular disease) [12]. The weight of the gland correlates with functionality and thus serum calcium levels [13]. There are only isolated case reports of non-functioning GPAs [14, 15]. Patients with GPA have higher mean preoperative PTH and serum calcium levels but are less likely to have symptoms of hypercalcemia [3]. The mechanism whereby they are asymptomatic remains unclear. This subset of asymptomatic patients will naturally present later thus these adenomas may grow to enormous dimensions before detection. The history of rapid onset in this case is unusual. We postulate that this was due to hemorrhage in the cystic component of the lesion. Such rapid enlargement would not otherwise be expected with a low MIB1 count. Ultrasonography may underestimate the size of these adenomas as only a proportion of the adenoma may be visualized transcervically [16].

Hungry bone syndrome (HBS) is the name given to the rapid severe decline in serum calcium due to the abrupt withdrawal of PTH following parathyroidectomy in patients with severe PHPT. Our patient developed HBS on the third postoperative day. The incidence of HBS in patients post-parathyroidectomy for PHPT was 13 % in one case series [17]. The volume of the gland resected correlates with the risk of HBS and is more predictive of HBS than other risk factors such as older age, preoperative blood urea nitrogen or alkaline phosphatase concentrations [17]. Treatment involves oral and/or intravenous administration of calcium and correction of magnesium. In our case the acute drop in phosphate level postoperatively resulted in cautious phosphate supplementation being administered orally from day 3 to 6 postoperatively. However, when reviewed by the endocrinology team this supplementation was discontinued on day 6, in keeping with the best practice guidelines of avoiding phosphate replacement in the setting of HBS as this may exacerbate hypocalcemia. Prophylactic measures such as preoperative vitamin D supplementation and preoperative bisphosphonates are currently being studied [18].

GPAs have both overlapping and distinguishing features when compared with parathyroid adenomas and carcinomas. GPAs are genetically similar to “usual” parathyroid adenomas with regards to frequent MEN1 mutations and infrequent hyperparathyroidism 2 gene (*HRPT2*) mutations. However, they are more akin to carcinoma in terms of loss of adenomatous polyposis coli (APC) immunoreactivity, parafibromin expression, and gain of chromosome 5 [12]. APC has the potential to become a valuable molecular screening tool that could help risk stratification of atypical parathyroid adenomas [19]. *HRPT2* gene and parafibromin staining are useful methods of detecting carcinoma when other tests reveal negative results and clinically carcinoma is a possibility. Unfortunately they are not available in our institution.

GPAs may mimic parathyroid carcinomas due to their large glandular size and extreme level of hypercalcemia. Endocrinologists have also questioned whether GPAs possess malignant potential [20]. Although follow-up time is relatively short, no recurrences have been reported [12]. Nor is there any increased incidence of persistent or recurrent hyperparathyroidism when compared to “regular-sized” parathyroid adenomas [3]. However, because of the molecular resemblance to carcinomas, patients with atypical adenomas should be followed up carefully.

## Conclusions

Acute PHPT with severe hypercalcemia is a rare and serious disorder [21, 22]. Any patient with an isolated severe hypercalcemia, particularly a patient as young as ours, warrants emergency admission and a thorough endocrinological work-up. Significantly elevated serum calcium is more suggestive of hypercalcemia of malignancy or of parathyroid carcinoma than of parathyroid adenoma. An intrathyroidal mass may be a GPA. Postoperatively, HBS is a serious and potentially avoidable condition. The clinician should be highly attuned to the risk of hypocalcemia following resection of GPA and maintain a low threshold for calcium and/or vitamin D supplementation.

## Abbreviations

APC: Adenomatous polyposis coli
BP: Blood pressure
eGFR: Estimated glomerular filtration rate
GPAs: Giant parathyroid adenomas
HBS: Hungry bone syndrome
HR: Heart rate
*HRPT2*: Hyperparathyroidism 2 gene
MEN1: Multiple endocrine neoplasia type 1
PHPT: Primary hyperparathyroidism
PTH: Parathyroid hormone

## References

[1] Wermers R, Khosla S, Atkinson E, Achenbach S, Oberg A, Grant C, et al. Incidence of primary hyperparathyroidism in Rochester, Minnesota, 1993-2001: an update on the changing epidemiology of the disease. J Bone Miner Res. 2006;21(1):171–7. 10.1359/JBMR.050910 16355286

[2] Yeh MW, Ituarte PH, Zou Hui C, Nishimoto S, Liu In-Lu A, Harari A. Incidence and prevalence of primary hyperparathyroidism in a racially mixed population. J Clin Endocrinol Metab. 2013;98(3):1122–9. 10.1210/jc.2012-4022 PMC3590475 23418315

[3] Spanheimer PM, Stoltze AJ, Howe JR, Sugg SL, Lal G, Weigel RJ. Do giant parathyroid adenomas represent a distinct clinical entity? Surgery. 2013;154(4):714–19. 10.1016/j.surg.2013.05.013 PMC3787983 23978594

[4] Yao K, Singer F, Roth S, Sassoon A, Ye C, AE G. Weight of normal parathyroid glands in patients with parathyroid adenomas. J Clin Endocrinol Metab. 2004;90(1):595. 10.1210/jc.2003-031184 15240594

[5] Takeichi N, Nishida T, Fujikura T, Hiraoka T, Wakabayashi T, Yotsumoto I. Two cases of large functioning parathyroid adenomas in atomic bomb survivors. Gan No Rinsho. 1983;29:851–4. 6876455

[6] Salehian M, Namdari O, Mohammadi S, Fazaeli YH. Primary hyperparathyroidism due to a giant parathyroid adenoma: a case report. Int J Endocrinol Metabol. 2009;9(2):101–5.

[7] Cakmak H, Tokat A, Karasu S, Ozkan M. Giant mediastinal parathyroid adenoma. Tuberk Toraks. 2011;59(3):263–5. 10.5578/tt.2419 22087523

[8] Power D, Kavanagh D, Hill A, O’Higgins N, McDermott E. Unusual presentation of a giant parathyroid adenoma: report of a case. Surg Today. 2005;35:235–7. 10.1007/s00595-004-2902-6 15772795

[9] McKay G, Ng T, Morgan G, Chen R. Giant functioning parathyroid cyst presenting as a retrosternal goitre. ANZ J Surg. 2007;77(4):297–304. 10.1111/j.1445-2197.2007.04037.x 17388841

[10] Shields T, Immerman S. Mediastinal parathyroid cysts revisited. Ann Thorac Surg. 1999;67(2):581–90. 10.1016/s0003-4975(99)00018-1 10197708

[11] Levin K, Galante M, Clark O. Parathyroid carcinoma versus parathyroid adenoma in patients with profound hypercalcemia. Surgery. 1987;101(6):647–60. 3589961

[12] Sulaiman L, Nilsson IL, Juhlin CHF, Höög A, Larsson C, Hashemi J. Genetic characterization of large parathyroid adenomas. Endocr Relat Cancer. 2012;19(3):389–407. 10.1530/ERC-11-0140 PMC3359501 22454399

[13] Moretz W, Watts T, Virgin F, Chin E, Gourin C, Terris D. Correlation of intraoperative parathyroid hormone levels with parathyroid gland size. Laryngoscope. 2007;117(11):1957–60. 10.1097/MLG.0b013e31813c14fc 17891053

[14] Diom E, Fagan J, Govender D. Giant cystic parathyroid adenoma masquerading as a retropharangeal abscess. Otolaryngology. 2014;4(1):154.

[15] Kiverniti E, Kazi R, Rhys-Evans P, Nippah R. Airway obstruction due to giant non-parathyroid hormone producing parathyroid adenoma. J Cancer Res Ther. 2008;4:197–9. 10.4103/0973-1482.44292 19052395

[16] Garas G, Poulasouchidou M, Dimoulas A, Hytiroglou P, Kita M, Zacharakis E. Radiological considerations and surgical planning in the treatment of giant parathyroid adenomas. Ann R Coll Surg Engl. 2015;97(4):64–6. 10.1308/003588415X14181254789682 PMC4473887 26263956

[17] Brasier A, Nussbaum S. Hungry bone syndrome: clinical and biochemical predictors of its occurrence after parathyroid surgery. Am J Med. 1988;84(4):654–60. 10.1016/0002-9343(88)90100-3 3400660

[18] Witteveen J, van Thiel S, Romijn J, Hamdy NA. Hungry bone syndrome: still a challenge in the post-operative management of primary hyperparathyroidism: a systematic review of the literature. Eur J Endocrinol. 2013;168(3):R45–53. 10.1530/EJE-12-0528 23152439

[19] Juhlin C, Nilsson I, Johansson K, Haglund F, Villablanca A, Höög A, et al. Parafibromin and APC as screening markers for malignant potential in atypical parathyroid adenomas. Endocr Pathol. 2010;21(3):166–77. 10.1007/s12022-010-9121-z 20473645

[20] Hundahl S, Fleming I, Fremgen A, Menck H. Two hundred eighty-six cases of parathyroid carcinoma treated in the U.S. between 1985–1995: a National Cancer Data Base Report. The American College of Surgeons Commission on Cancer and the American Cancer Society. Cancer. 1999;86(3):538–44. 10.1002/(sici)1097-0142(19990801)86:3<538::aid-cncr25>3.0.co;2-k 10430265

[21] Dogan U, Koc U, Mayir B, Habibi M, Dogan B, Gomceli I, et al. Life-threatening intrathyroidal parathyroid adenoma. Int J Clin Exp Med. 2015;8(1):1501–3. PMC4358619 25785164

[22] Neagoe RM, Sala DT, Borda A, Mogoanta CA, Muhlfay G. Clinicopathologic and therapeutic aspects of giant parathyroid adenomas – three case reports and short review of the literature. Rom J Morphol Embryol. 2014;55(2 Suppl):669–74. 25178342

